# Invited Commentary: Evaluating Nationwide Application of Minimally Invasive Surgery for Treatment of Small Bowel Neuroendocrine Neoplasms

**DOI:** 10.1007/s00268-021-06088-2

**Published:** 2021-05-15

**Authors:** Per Hellman, Peter Stålberg

**Affiliations:** Uppsala University, University Hospital, SE75185 Uppsala, Sweden

In the paper by Kacmaz et al., the concept of performing laparoscopic surgery in small intestinal neuroendocrine tumors (SI-NETs) was studied, by using a national registry from the Netherlands [1]. Thus, 492 patients underwent surgery between 2005 and 2015 for SI-NET; of these, 29% were performed laparoscopically (*n* = 140). The two groups differed since the open surgery group had significantly more multifocal tumors resected, more positive lymph nodes and more often stage IV disease. These facts indicate that the individuals operated with open surgery had more advanced disease than the ones operated on laparoscopically, which indeed is confirmed by the shorter overall survival (5-year OS 71% vs. 84%, *p* = 0.004). The authors conclude that laparoscopic surgery for SI-NET is feasible for the more favorable patients.

The questions raised when discussing laparoscopic procedures in SI-NET are: (1) Will all of the often multiple, primary tumors be discovered? In open surgery, palpation of the entire bowel is mandatory in order to find all primaries. How is this performed laparoscopically? (2) Is it possible to perform a safe mesenteric dissection, especially when lymph node metastases are situated proximally? (3) Is the, often fibrotic, shortening of the mesentery a technical problem at laparoscopy? (4) Is there a similar radicality between open and laparoscopic resections; similar levels of reached R0 resections?

Before looking into these entities in detail, one must also consider the indication for surgery. In other diagnoses, the introduction of laparoscopic surgery may have widened the surgical indication, sometimes unnecessarily, leading to increased risks and unnecessary operations (cholecystectomy, pancreatic resections), and sometimes offering far less complications and better patient outcome (adrenalectomy, prostatectomy). Laparoscopic bowel surgery has been used for rectal and colon cancer with success, also offering a less traumatic operation without reducing the yield of positive lymph nodes or increasing positive margins. In fact, many witness a more secure surgery by using the magnification obtained by the camera, and after an initial learning curve it is now possible to perform most laparoscopic procedures faster and more safely than many open procedures. In terms of small bowel surgery, laparoscopic procedures are rarer. Case series describe emergency surgery due to obstruction, perforations, etc. The indication for surgery in SI-NET should be either to reach R0 (absent or only resectable liver metastases present) or to reduce symptoms. The latter may be in an emergency situation when mechanical obstruction is present or semi-emergent due to long-lasting, continuous and intractable abdominal pain. In this paper, we see two different types of patients; the first is represented by a fairly stable patient, usually placed on somatostatin analogues, often with limited extent of disease (since R0 is reachable), while the second has an affected bowel (dilated, venous stasis, fibrotic adhesions, etc.) leading to abdominal symptoms. There is a third group that during the decades has been operated upon, which contain the stage IV patients, without possibility to reach R0 and without abdominal symptoms. Most studies today suggest that these patients should undergo “watchful waiting” rather than up-front surgery.

Thus, the perfect patient for laparoscopic surgery would be the patient where R0 is reachable, which according to recent studies preferably would be the patient without abdominal symptoms. Of course, some patients with abdominal symptoms may also be laparoscopically operated, but in this group of patients one may foresee a higher degree of conversion to open surgery. The reasons for this may be complicated adhesions, fragile bowel, proximally situated lymph node metastases with mesenteric vessel involvement, all of these being more present in patients with abdominal symptoms.

Technically, the authors of the current article have convincingly demonstrated that in patients where it is possible to dissect the small intestinal bowel (and sometimes the right colon) from the posterior abdominal attachments, one may exteriorize the bowel and perform the manual palpation in order to identify possible multiple primary tumors. In conjunction with this, the intraoperative survey of position and number of metastatic mesenteric lymph nodes is also possible. The following resection and new anastomosis may well be performed on the exteriorized bowel, which then is reinserted, and the small incision closed. Indeed, this small incision may also be performed initially in the procedure for the introduction of a hand-port device, in order to even further assist in the initial laparoscopic dissection.

The laparoscopic technique for SI-NET surgery is here to stay, but it is of crucial importance to not reduce our high standard of care in being complete in identifying all primary tumors and carefully balancing the length of resected bowel and removal of mesenteric metastases as to not inadvertently causing short bowel. Indeed, smaller solitary liver lesions may also be laparoscopically resected simultaneously.
